# Common mycorrhizal network: the predominant socialist and capitalist responses of possible plant–plant and plant–microbe interactions for sustainable agriculture

**DOI:** 10.3389/fmicb.2024.1183024

**Published:** 2024-03-26

**Authors:** Asad Ullah, Danmei Gao, Fengzhi Wu

**Affiliations:** ^1^Department of Horticulture, Northeast Agricultural University, Harbin, China; ^2^Key Laboratory of Biology and Genetic Improvement of Horticultural Crops (Northeast Region), Ministry of Agriculture and Rural Affairs, Northeast Agricultural University, Harbin, China

**Keywords:** AMF, rhizosphere, CMNs, root exudates interplant signaling, donor plant

## Abstract

Plants engage in a variety of interactions, including sharing nutrients through common mycorrhizal networks (CMNs), which are facilitated by arbuscular mycorrhizal fungi (AMF). These networks can promote the establishment, growth, and distribution of limited nutrients that are important for plant growth, which in turn benefits the entire network of plants. Interactions between plants and microbes in the rhizosphere are complex and can either be socialist or capitalist in nature, and the knowledge of these interactions is equally important for the progress of sustainable agricultural practice. In the socialist network, resources are distributed more evenly, providing benefits for all connected plants, such as symbiosis. For example, direct or indirect transfer of nutrients to plants, direct stimulation of growth through phytohormones, antagonism toward pathogenic microorganisms, and mitigation of stresses. For the capitalist network, AMF would be privately controlled for the profit of certain groups of plants, hence increasing competition between connected plants. Such plant interactions invading by microbes act as saprophytic and cause necrotrophy in the colonizing plants. In the first case, an excess of the nutritional resources may be donated to the receiver plants by direct transfer. In the second case, an unequal distribution of resources occurs, which certainly favor individual groups and increases competition between interactions. This largely depends on which of these responses is predominant (“socialist” or “capitalist”) at the moment plants are connected. Therefore, some plant species might benefit from CMNs more than others, depending on the fungal species and plant species involved in the association. Nevertheless, benefits and disadvantages from the interactions between the connected plants are hard to distinguish in nature once most of the plants are colonized simultaneously by multiple fungal species, each with its own cost-benefits. Classifying plant–microbe interactions based on their habitat specificity, such as their presence on leaf surfaces (phyllospheric), within plant tissues (endophytic), on root surfaces (rhizospheric), or as surface-dwelling organisms (epiphytic), helps to highlight the dense and intricate connections between plants and microbes that occur both above and below ground. In these complex relationships, microbes often engage in mutualistic interactions where both parties derive mutual benefits, exemplifying the socialistic or capitalistic nature of these interactions. This review discusses the ubiquity, functioning, and management interventions of different types of plant–plant and plant–microbe interactions in CMNs, and how they promote plant growth and address environmental challenges for sustainable agriculture.

## Introduction

The exploration of various interactions between members of the same or distinct kingdoms is of paramount importance for ecological stability, nutrient cycling, and the efficient management of an ecosystem. Most land plants are associated with mycorrhizal fungi for their nutritional demand, development, and increased resistance to stress (Compant et al., 2019; Bhatt et al., 2020). The history of interactions between plant–plant and plant–microbes is as old as plant colonization on Earth. In both natural and agricultural ecosystems, the invaded interactions may be positive or negative, depending on the mode of interest (Kuebbing and Nuñez, 2015). As such, plants make the obligatory interactions necessary for their existence by suppressing the growth of others or by sharing resources for the benefit of each other. The improvement of seedlings (Seiwa et al., 2020), impact on the plant and microorganism community (Kadowaki et al., 2018; Teste et al., 2020), activation of plant defense responses (Babikova et al., 2013; Song et al., 2014), and interplant nutrition (Bücking et al., 2016; He et al., 2019; Fang et al., 2021) are among the most specific responses regulated by such interactions. Subsequently, the release of volatile compounds and the transfer of essential mineral nutrients to plants *via* mycorrhizal fungi are good examples of positive plant–plant and plant–microbe interactions, respectively (Alaux et al., 2021; Ohsaki et al., 2022). Mycorrhizal fungi improve plant nutrient uptake and receive plant carbohydrates, interacting for the net benefit of both parties (Bücking et al., 2016; Gilbert and Johnson, 2017). On the flip side, the mechanism of allelopathy and necrotrophy in the colonizing plants caused by saprophytic microbes is an example of negative interactions (Dicke and Baldwin, 2010; Friedman, 2017). Most of these interactions affect the survival and behavior of connected plants and potentially influence competitiveness patterns at local and regional scales (Bücking et al., 2016). Therefore, deciphering these interactions would greatly improve our understanding of how these interactions affect terrestrial ecosystems and the potential for feedback on global change.

**Figure 1:**
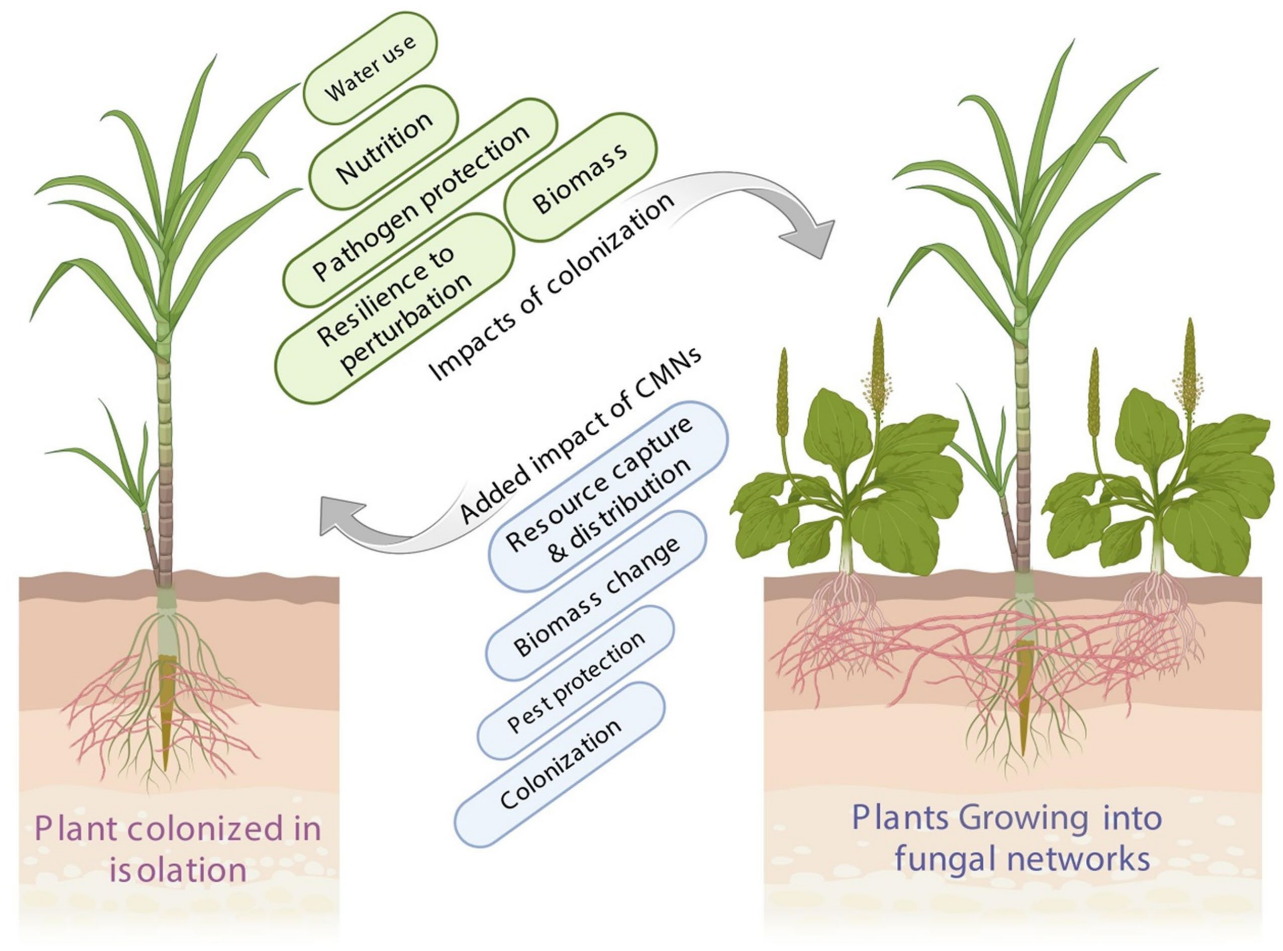
Impacts of plant integration into CMN and mycorrhizal colonization. Plants integrated into CMNs can experience enhanced nutrient acquisition and absorption, improving their overall growth and survival. Mycorrhizal colonization plays a vital role in increasing a plant’s resistance to various environmental stresses and toxic substances. CMNs facilitate interplant communication, enabling plants to exchange signals and respond to changes in the environment, leading to improved ecosystem resilience and biodiversity.

AMF are ancient fungal organisms that engage in mutualistic symbiotic relationships with the vast majority of land plant species for nutritional exchange (Figure 1). AMF improve plant nutrition by accessing nutrient sources that are otherwise inaccessible to roots (Wipf et al., 2019; Andrino et al., 2021). The great majority of AM fungi build extensive colonization networks with numerous neighboring plants for their carbon or nutritional supplies through CMNs, which are not host-specific (Rhodes, 2017; Simard, 2018; He et al., 2019; Bacha et al., 2023). CMNs play a crucial role in plant–plant interactions by generating warning signals and activating defense information (Song et al., 2015; Gilbert and Johnson, 2017; Oelmüller, 2019). Thus, evidence supports the multifunctionality of CMNs, which are involved in different types of AM interactions across ecosystems (Gilbert and Johnson, 2015).

**Figure 2:**
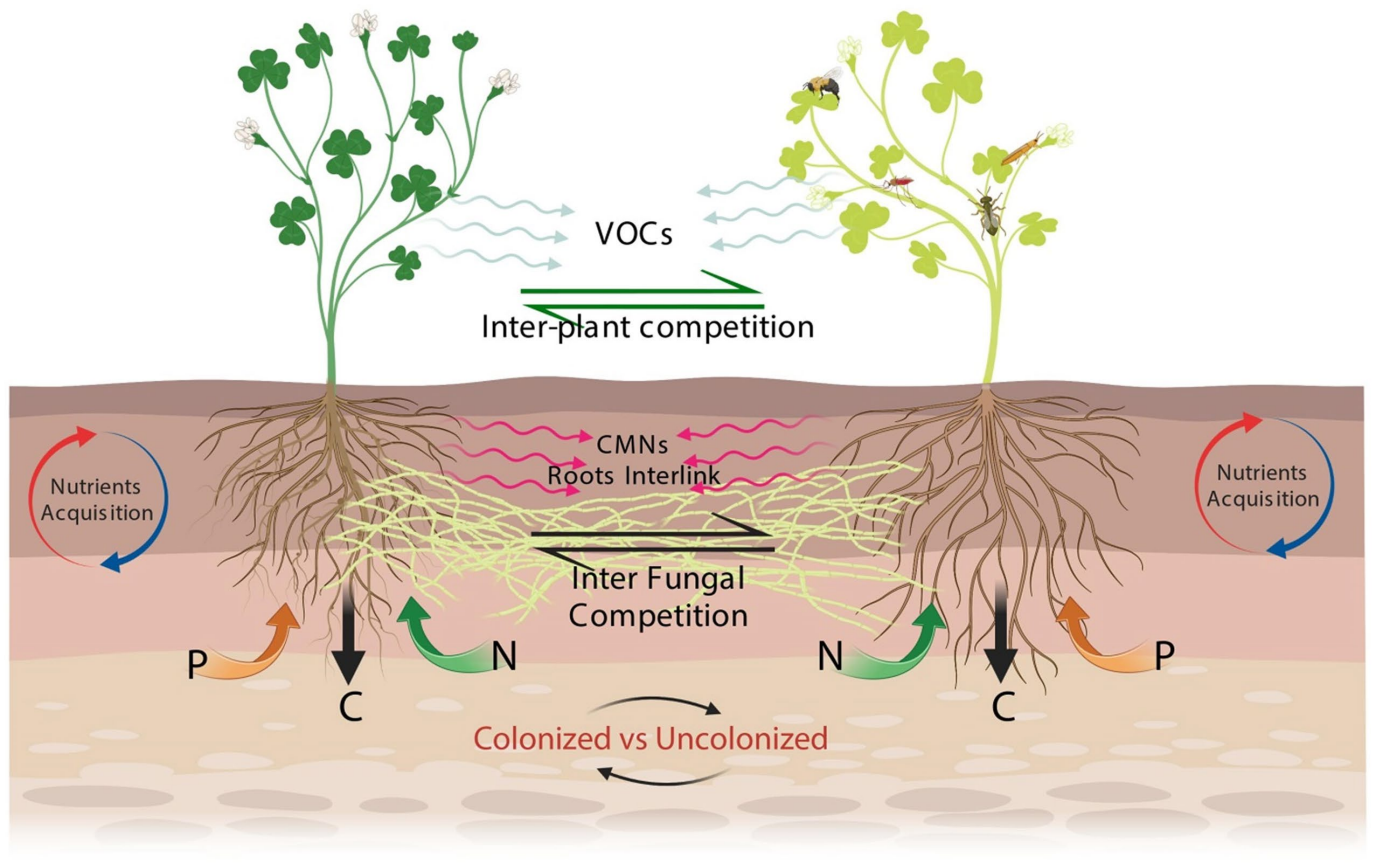
Schematic diagram of interplant signaling *via* CMNs in plant–plant interaction. In this mutualistic association, the roots of both plants are colonized by hyphal threads extending from the fungal colonization within their roots. Through this interconnected network, plants transmit signals, which can be chemical compounds like hormones or volatile organic compounds (VOCs), in response to stimuli or environmental changes (Rasheed et al., 2023). These signals travel through the CMNs *via* the mycorrhizal hyphal threads, spanning the soil and allowing for communication between the plants. The signals move from one plant to another through the interconnected hyphal threads. The roots of the receiving plant detect and receive these transmitted signals through specific receptors present in their root systems (Abdul Malik et al., 2020). These receptors recognize the signals emitted by the sending plant and initiate a response. The response may involve various physiological, biochemical, or molecular changes, such as alterations in growth patterns, nutrient uptake, defense mechanisms, or adaptive behaviors. This interaction between the plants creates a feedback loop where both plants can generate signals that are transmitted through the CMNs, allowing for reciprocal communication and response. This process underscores the vital role of the CMNs and mycorrhizal fungi in facilitating interplant signaling and promoting plant–plant interactions in natural environments.

Similarly, CMNs can actively participate in improving plant resistance and tolerance to abiotic stress (Plouznikoff et al., 2016; Bacha et al., 2023). Recent research indicates that CMNs influence the survival, fitness, behavior, and competitiveness of numerous fungal and plant species that interact and “communicate” *via* these networks. CMNs enable the fungus to establish connections with several trade partners, ensuring a consistent carbon supply for the fungus (Fellbaum et al., 2014). This is particularly important when one host plant becomes unable to transmit resources to its fungal partner owing to disease, herbivore damage, or premature senescence (Figure 2). From an ecosystem perspective, exploring the possible interactions of plant–plant and plant–microbes mediated by CMNs for nutritional strategies is a critical component for enhancing ecosystem services effectively. To date, numerous studies have been conducted to reveal these interaction processes; however, many paradoxical results still exist, and the debate about these issues has never ceased so far. Thus, it is of paramount importance to shed light on the most recent advances in literature and highlight the potential research question gaps for guiding the upcoming studies in the specified areas.

## The common mycorrhizal networks

The CMNs are an underground network of hyphae created by mycorrhizal fungi that join with plant roots to form a symbiotic relationship (Bonneau et al., 2019; Deja-Sikora et al., 2019; Figueiredo et al., 2021). The network connects individual plants together, allowing for the transfer of water, carbon, nitrogen, and other nutrients and minerals between participants (Wipf et al., 2019; Rog et al., 2020). CMNs are involved in the long-distance transport of carbon and micro-nutrients (Teste et al., 2015). Similarly, macro-nutrients such as N and P were transferred through CMNs (Muneer et al., 2020). A study conducted by Gao et al. (2021) pointed out that CMNs facilitate interspecific interactions between tomato and potato–onion to enhance K uptake and boost tomato growth. It is believed that plant species can interact and communicate *via* these CMNs (Gorzelak et al., 2015; Pickles et al., 2017; He et al., 2019). Among the reported effects of such connectivity are the improvement of seedling establishment (Bingham and Simard, 2011; Seiwa et al., 2020), impact on plant and microorganism community compositions (Teste et al., 2015; Kadowaki et al., 2018), induction of plant defense responses (Babikova et al., 2013; Song et al., 2014), plant communication through a variety of phytohormones such as jasmonic acid, methyl jasmonate, and zeatin riboside (Song et al., 2010), and nutrient exchange, which may play a pivotal role for interplant nutrition (Bücking et al., 2016; He et al., 2019; Fang et al., 2021).

The network can enhance mycorrhizal colonization of plant species and improve their growth and performance (Muneer et al., 2020). Mycorrhizal networks have existed for over 400 million years, and up to 90% of all land plants participate in these networks (Vanishri and Khandappagol, 2018; Hatté et al., 2020; Hawkins et al., 2023). Mycoheterotrophic plants rely on carbon transfer from mycorrhizal networks as their main source of energy (Watkinson, 2016; Hatté et al., 2020). The formation of CMNs is facilitated by the presence of mycorrhizal fungi in the soil. These fungi form relationships with the roots of multiple plants, allowing for the creation of a network of hyphae that link the plants together (Karst et al., 2023). In addition to transferring nutrients, carbon, and other resources, the ability of plants to alter their behavior based on the signals or cues they receive from other plants through the CMNs highlights their important role in plant communication (Kytöviita et al., 2003; Weremijewicz and Janos, 2019).

The taxonomical framework of CMNs can vary depending on the specific fungus species involved. AMF and ectomycorrhizal fungi (ECM) are two main types of mycorrhizal networks and are known to facilitate the formation of CMNs and can connect different plant species together (Teste et al., 2020). The functioning of CMNs is not solely based on ectomycorrhizal fungi (ECM), but can also involve AMF in various plant species (Gorzelak et al., 2015; Liang et al., 2020, 2021). The formation of these networks is also dependent on the plant species involved. Different plant species may have varying levels of dependency on AMF for nutrient exchange and therefore may form CMNs with different fungal species (Pringle et al., 2009; Simard et al., 2015). In general, the hyphae of mycorrhizal fungi facilitate nutrient exchange between plants linked together in CMNs (Muneer et al., 2020). Therefore, the taxonomy of CMNs cannot be described by a simple framework and is dependent on the specific context of the fungal and plant species involved (van der Heijden et al., 2015; Authier et al., 2022; Kuyper and Jansa, 2023).

## CMNs in dominant plant–plant interactions

AMF are important mutualistic microorganisms that can connect the roots of plants to form CMNs. In plant–plant interactions, CMNs play a major role by interlinking the root systems of two or more host plants (Simard et al., 2012; Hoeksema, 2015). It allows distant plant individuals to communicate and help each other (Gilbert and Johnson, 2017). Although carbon and mineral nutrients can move through CMNs, our understanding of factors controlling the direction, speed, and magnitude of these movements is still elusive.

Plant–plant interactions may be socialist or capitalist (Van Der Heijden and Horton, 2009; Kuebbing and Nuñez, 2015). In the socialist network, resources are distributed more evenly, providing benefits for all connected plants, such as symbiosis. For example, direct or indirect transfer of nutrients to plants, direct stimulation of growth through phytohormones, antagonism toward pathogenic microorganisms, and mitigation of stresses (Bücking et al., 2016; Gilbert and Johnson, 2017). For the capitalist network, AMF would be privately controlled for the profit of certain groups of plants, hence increasing competition between connected plants. Such plant interactions invading by microbes act as saprophytic and cause necrotrophy in the colonizing plants (Friedman, 2017). Depending on the resource distribution, plants may either benefit from an equal distribution of resources or from increased discrepancies in resources. In the first case, excess nutritional resources may be donated to the receiver plants by direct transfer (He et al., 2019; Fang et al., 2021). In the second case, unequal distribution of resources occurs, which certainly favors individual groups and increases competition between interactions (Wipf et al., 2019). Tomato benefited more from intercropping with potato–onion in terms of AMF abundance, root colonization, and P absorption (Gao et al., 2021). Taken into account, the mode of interplant connection might have evolutionary consequences for CMNs, leading to ecosystem-wide impacts (Gorzelak et al., 2015). This largely depends on which of these responses is predominant (“socialist” or “capitalist”) at the moment plants are connected.

AMF acquire bargaining power when they can differentiate between host plants in their CMNs. The phenomenon of natural selection should favor fungi that possess the ability to form CMNs with a diverse array of host plants because competition among plants would force the competing plant to allocate a higher amount of carbon toward their fungal partner to receive a larger proportion of nutrients from the CMNs (Bücking et al., 2016).

To predict how the co-existence of plant species in the ecosystem is stabilized, many different theories have been proposed in light of all the possible effects of CMNs. On one side, the biological market theory is based on the idea that fungi may identify the ideal plant partner and reassign nutrients in accordance with the plant’s carbon (C) gain (Weremijewicz et al., 2016). It is also called “reciprocal rewards” mechanisms, i.e., the more nutrients an AMF provides to a plant, the more carbon it receives in return (Werner and Kiers, 2015; Wang et al., 2019). On the flip side, we have the source-sink theory, in which the resource migrates along a concentration gradient, which might lead to a more equitable distribution of resources among network partners (Montesinos-Navarro et al., 2017; Muneer et al., 2020). It is believed that interplant resource exchanges are regulated by source-sink; as such, they can form the relationship between donor and receiver. For instance, the nutrient-rich plants provide minerals to the neighboring plants, which are nutrient deficient (Simard et al., 2012).

Fungus is said to be the “natural internet” of the earth. CMNs are information superhighways that expedite interactions between vast and diverse populations of individuals. It enables diverse individuals to collaborate and share information (Gilbert and Johnson, 2017). Fungal networks also improve host plant immunity. Fungi are responsible for the colonization of plant roots, which triggers the production of chemical substances associated with plant defense. The mechanism known as “priming” helps the immune system respond more quickly and efficiently. Simply connecting to mycelial networks makes plants more disease-resistant. Such connectivity is associated with the improvement of seedling establishment (Bingham and Simard, 2011; Seiwa et al., 2020), influencing plant make-up and soil microbial communities (Kadowaki et al., 2018; Wipf et al., 2019; Sharifi and Ryu, 2021), stimulating plant defense responses (Babikova et al., 2013; Song et al., 2014), plants communicating with each other through various phytohormones, including jasmonic acid, methyl jasmonate, and zeatin riboside (Song et al., 2010), nutrient exchange between plants has been identified as a potentially important mechanism for interplant nutrition, as highlighted by Bücking et al. (2016), He et al. (2019), and Fang et al. (2021). Enhanced soil physicochemical qualities and the provision of limited nutrients, particularly nitrogen (N), to both the donor and recipient plants are two factors that contribute to the potential enhancement of plant growth *via* the use of CMNs (Muneer et al., 2020).

Mycorrhizal hyphae, either individually or in the form of rhizomorph clusters that resemble chords, have the potential to form external bridges between the root tips of the same plant and interconnect distinct plants below ground (Smith and Read, 2010). It has been reported that the internal hollow space within rhizomorphs may serve as a conduit for the transport of biogenic volatile organic compounds (BVOCs) (Barto et al., 2012). Furthermore, the presence of fungi in the rhizosphere not only serves as a physical mode of transportation across the hyphae but also influences the soil structure and sustains soil aggregates, thereby facilitating the movement of root exudates and even below-ground BVOCs through the soil matrix (Barto et al., 2012; Paudel et al., 2016). The healthy bean plants exhibited varying levels of BVOC emission in response to their connection with aphid-infested bean plants through CMNs, thereby attracting a natural enemy of aphids (Babikova et al., 2013). In contrast, bean plants that were not connected by CMNs to aphid-infested bean plants did not show any statistically significant alterations in BVOC emissions (Babikova et al., 2013). It has been shown that BVOC emissions from receiver plants are modulated by stress signals transmitted across CMNs.

In previous research, it has been found that receiver plants exhibited an upregulation in the expression of diverse genes associated with defense mechanisms. The proteins examined in this study encompass a group of pathogen-related proteins: PR1, PR-2, PR-3, phenylalanine ammonia-lyase (PAL), lipoxygenase (LOX), lipoxygenase D (LOXD), allene oxide cyclase (AOC), PPD, and SOD, as well as two serine protease inhibitors (PI-I and PI-II) (Song et al., 2010, 2014, 2015). The receiver plant exhibited an upregulation in the enzymatic activity within a 24-h period, which closely aligned with the timeframe observed in the donor plants and significantly exceeded the rate of carbon transfer across the CMNs (Song et al., 2014, 2015). It is postulated that in the context of stressful events, the primary factors that pass through CMNs from herbivore-infested donor plants are stress signals rather than nutrients. The jasmonic acid pathway is an extensively studied biological pathway that plays a vital role in responding to tissue injury and activating defense mechanisms against herbivores. LOX and AOC enzymes possess notable significance (Howe and Jander, 2008). Based on the available limited literature, it is reasonable to expect that defense upregulation in uninfested “receiver” plants linked *via* CMNs to infested “donor” plants will be quite similar.

Plant hormones mediate signaling within plants, aid in the establishment of “synapses” between root-mycorrhizal fungi, and pass through CMNs, making them an ideal candidate for signaling (Boyno and Demir, 2022). There is still some doubt about whether hormones play a significant role in stress signaling from a donor plant to a receiver plant across the CMNs. Researchers have used stable isotope labeling to examine nutrient sharing across CMNs (He et al., 2009). In meticulously planned experiments, synthetic hormones that are labeled with stable isotopes have the potential to be utilized for investigating the involvement of plant hormones in stress signaling across CMNs. There is a high probability that the stress signals transmitted by CMNs do not involve the transport of molecules across the root-mycorrhizal fungi “synapse.” It is speculated that stress signals originating from the emitter are thought to act as stimuli, triggering the production of infochemicals in the CMNs, which are then detected by the receiver plant. In this case, the utilization of isotope labeling techniques may not provide insights into the underlying mechanisms of stress signaling. Secondary compounds have shown great potential for explaining the complex below-ground plant signaling network (Hong et al., 2022). A study encompasses the examination of non-targeted metabolites present in the root exudates and secondary compounds of the CMNs, which are hypothesized to play a role in signal transmission. This study has the potential to offer valuable insights into the composition and properties of these signaling compounds. At the moment, studying the signaling molecules that travel through the CMNs is hindered by the fact that we are unable to separate secondary metabolites from fungal hyphae.

In natural ecosystems, mycorrhizal plant networks also form commensalistic and even antagonistic interactions relative to the mutualistic interactions that occur between connected plants (Toju et al., 2013). Therefore, depending on the fungi and plants engaged in the interaction, some plant species may benefit from CMNs more than others. Allelopathy is a good example of antagonistic interactions between plants in which a subsequent toxic chemical is secreted by the plants to inhibit their growth or to kill the competing plant (Friedman, 2017). Numerous plants have allelopathic characteristics; a phytotoxin compound (gallic acid) was released by the invasive plant *P. australis* to restrict plants in neighboring areas (Rudrappa et al., 2009). Similarly, Bieberich et al. (2018) pointed out that 2-methoxy-1,4-naphthoquinone (2-MNQ) not only potentially negatively affects native plants but also the interplant from the same species in neighbors (autotoxicity).

Plants need mycorrhizae fungus for long-distance transmission of plant signals and allelochemicals. The plant species *Tagetes tenuifolia* emits two types of allelopathic chemicals: a lipophilic called thiophene and a hydrophilic called imazamox. These chemical substances have the ability to diffuse over a distance of more than 12 cm *via* the common mycorrhiza network, as shown by Barto et al. (2011). Mycorrhizae fungus has the unique ability to transmit defensive signals from stressed plants to their healthy neighbors. According to Song et al. (2010), 18 h after inoculation, tomato plants that have been infected with the necrotrophic pathogenic fungus *Alternaria solani* are able to transmit a signal via mycorrhizae hypha to neighboring plants that have not been infected. The relative expression levels of defense-related genes such as PAL, lipoxygenase, polyphenol oxidase, and pathogenesis-related proteins (PR1, PR2, and PR4) in the recipient plants were the same as in the donor plants. Alaux et al. (2020) designed a complex *in vitro* experiment to further establish the importance of mycorrhizae in plant–plant signal transduction. The results demonstrated that a tomato plant infected with *Phytophthora infestans* could convey a signal to its healthy neighbors *via* mycorrhizae hypha to induce the transient expression of JA-related genes.

The underground connectivity of plants through CMNs potentially affects both source-sink and market theories. For instance, the vast hyphal network allows their host plant to interact underground with several plants at a time to efficiently absorb resource nutrients (Figure 1). Also, these underground hyphae have the capability to enter plant roots at various points of entry for the trade of nutrients with plants (Johnson and Gilbert, 2015). Thus, CMNs act as mediators in most of the interactions between plants and play an important role in nutrient facilitation, transportation of water stress signals, and allelochemicals in natural ecosystems (Figure 1; Gorzelak et al., 2015; Teste et al., 2015). In CMNs, the better carbon provider is the targeted species once the targeted CMNs constitute the strongest nutrient sink compared with the weaker carbon source (Weremijewicz et al., 2016). The biological market theory is a valuable paradigm for investigating how collaboration might be sustained in plant–plant and plant–microbe interactions. The idea contends that resource trade can be evaluated from an economic perspective: partners on both sides of the interaction compete, and those who offer the best “rate of exchange” are favored (Werner et al., 2014). As such, both plants and fungi are allowed to select the quality and quantity of the resources supplied by their partner to adjust their own resource allocation (Werner and Kiers, 2015; Wang et al., 2019). All these studies suggest that CMNs are probably ubiquitous, although the mechanisms of the interactions between plants through CMNs have been under debate (Fellbaum et al., 2012; Bücking et al., 2016). Therefore, understanding the complex relationships among plant species and their dependency on soil nutrient properties is of paramount importance for the better optimization of an ecosystem.

Furthermore, CMNs are responsible for transporting below-ground warning signaling to neighboring interconnected plants to provoke the defense system (Babikova et al., 2013; Song et al., 2014). In particular, the defense response induced against insect herbivores, herbivory-elicitors, and hemibiotrophs (Alaux et al., 2020) leads to the emission of protective volatile signals to repel subsequent herbivores from the plant. Assessing the threat, CMNs transferred the subsequent signal to the interlinked plants to induce a similar defense response. This signal might be likely transferred further to an indirectly interconnected neighboring plant and so on for the potential future threat. As such, if the attack occurs, the plant will respond faster and more strongly. It has been suggested that transmitted signals sent by interconnected plants can travel through CMNs of at least 20 cm in length (Babikova et al., 2013). A study conducted by Song et al. (2014) on tomato plants pointed out that CMNs are the signaling pathways between plants during an attack by caterpillars. This phenomenon works when an herbivore attacks infested plants (donors) and transmits warning signals to neighboring non-infested plants through CMNs (Figure 2). The non-infested plants (receivers) upregulate the genes of defense in response to these transmitted signals (Song et al., 2019). The cytoplasmic streaming within hyphae, capillary action, root exudates, amino acids, microorganisms, and conduits for wound-induced electrical impulses are some of the potential processes behind the transfer of signals with CMNs (Johnson and Gilbert, 2015). Plant responses are also related to the production of ethylene and salicylic acid (Zhang et al., 2019). Interestingly, only a particular aspect of the JA response was activated in the priming of plant defense leading to a limitation on the potential cost of induced defense. To reduce pest-related crop losses and effectively use this approach, more research is needed to investigate additional elements in field conditions, notably the potential links between different CMNs and the putative relay of signals among plants (Wipf et al., 2019). Also, the leading phenomenon of signaling in nature remains largely puzzling, especially in terms of the signal transfer mechanism. Therefore, the prediction of ecosystem dynamics among connected plants is complex and still a huge challenge.

Johnson and Gilbert (2015) found three potential pathways for CMN interplant signaling mechanisms:

1. Capillary action or microorganisms both contribute to the movement of molecules in liquid films on the exterior surface of hyphae.
2. The transportation of signal molecules by the process of cytoplasmic streaming within hyphae.
3. Electrical signals triggered by a wound.

Due to the intimate contact of fungal hyphae with soil particles, which makes the transfer of water inefficient, mechanism 1 is very unlikely to occur. It is therefore highly unlikely that signals can be transferred over long enough distances and at fast enough speeds to prevent herbivores from invading neighboring plants *via* mechanism 1. It would make sense for cytoplasmic streaming within hyphae to be another possible pathway for interplant signal transfer. Sugars, amino acids, and lipids are known to be exchanged between mycorrhizal fungi and plants (Smith and Read, 2010). This signal pathway might easily be extended from plant–fungus to plant–fungus–plant. CMN-mediated interplant signaling is most likely supported by mechanism 2, according to Johnson and Gilbert (2015).

Recent research by Thomas and Cooper (2022) finds it rather interesting that electrical signals can serve as a medium for mycelium-mediated interplant signal transfer. Mycorrhizal-inoculated *Pisum sativum* and *Cucumis sativus* seedlings were cultivated on agar plates. A narrow space was observed between the agar plates, prompting the mycelium to bridge this gap. This ensured that an electrical signal would pass through the mycelium rather than the agar. The leaves of donor plants were clipped, which caused damage to the leaves and triggered an electrical response in the donor. This electrical signal could successfully transmit from the donor to the recipient plant *via* the mycelium bridge. Thomas and Cooper (2022) conducted an experiment to successfully demonstrate that electrical signals can be transmitted from one plant to another through CMNs. Nevertheless, the researchers did not evaluate any additional responses in the receiver plants apart from the electrical signal transduction. Future research endeavors to integrate the methodologies of Song et al. (2015) and Thomas and Cooper (2022) in order to assess both the defense response and electrical signal transduction in receiver plants.

In an alternative scenario, the signal could be made up of various substances that are transmitted between plants through the means of CMNs. According to Rasheed et al. (2023), it is possible that signal molecules transmitted across CMNs may not exhibit the ability to traverse from the root to the fungus. These molecules may stimulate the fungus to generate its own signal, which is subsequently detected by the receiver plant. The specific nature and mechanisms underlying signal transmission in plants and fungi have yet to be fully elucidated, although several potential candidates have shown promising indications. The jasmonate pathway is a potential mechanism involved in the transduction of defense signals in plants. The pathway encompasses a diverse array of signaling molecules referred to as jasmonates, which play a key role in regulating wound responses in plants. A study conducted by Song et al. (2014) observed that donor plants, which possessed a mutation preventing the synthesis of jasmonates, exhibited an inability to induce defense responses in receiver plants. On the flip side, donors with functional jasmonate pathways exhibited the capability to induce defense mechanisms. The study conducted by Song et al. (2014) did not provide conclusive evidence regarding the transfer of jasmonates *via* CMNs. However, it is important to note that Rasheed et al. (2023) propose that this possibility should still be considered. The exact mechanism by which the fungal signal travels within the Common Mycelial Network (CMN) remains unclear.

The current literature indicates that the primary mechanism for interplant signal exchange through CMNs is chemical transfer, facilitated by cytoplasmic streaming within hyphae. This phenomenon can be attributed to the extensive research conducted on this particular form of transfer within the context of mycorrhizal associations. While the involvement of the jasmonate pathway in signal transmission within plants is well established, there remains uncertainty regarding the ability of jasmonates to travel through CMNs. The alternative action potential mechanism has received comparatively less attention in research compared to chemical transfer mechanisms. However, based on the existing knowledge of both mechanisms, it appears that they have an equal likelihood of contributing to interplant signaling, at least to some extent. In contrast, it is possible that the two mechanisms are not mutually exclusive and may both play a role in facilitating interplant signal transfer (Gilbert and Johnson, 2017).

## Dominant plant–microbe interactions

**Figure 3:**
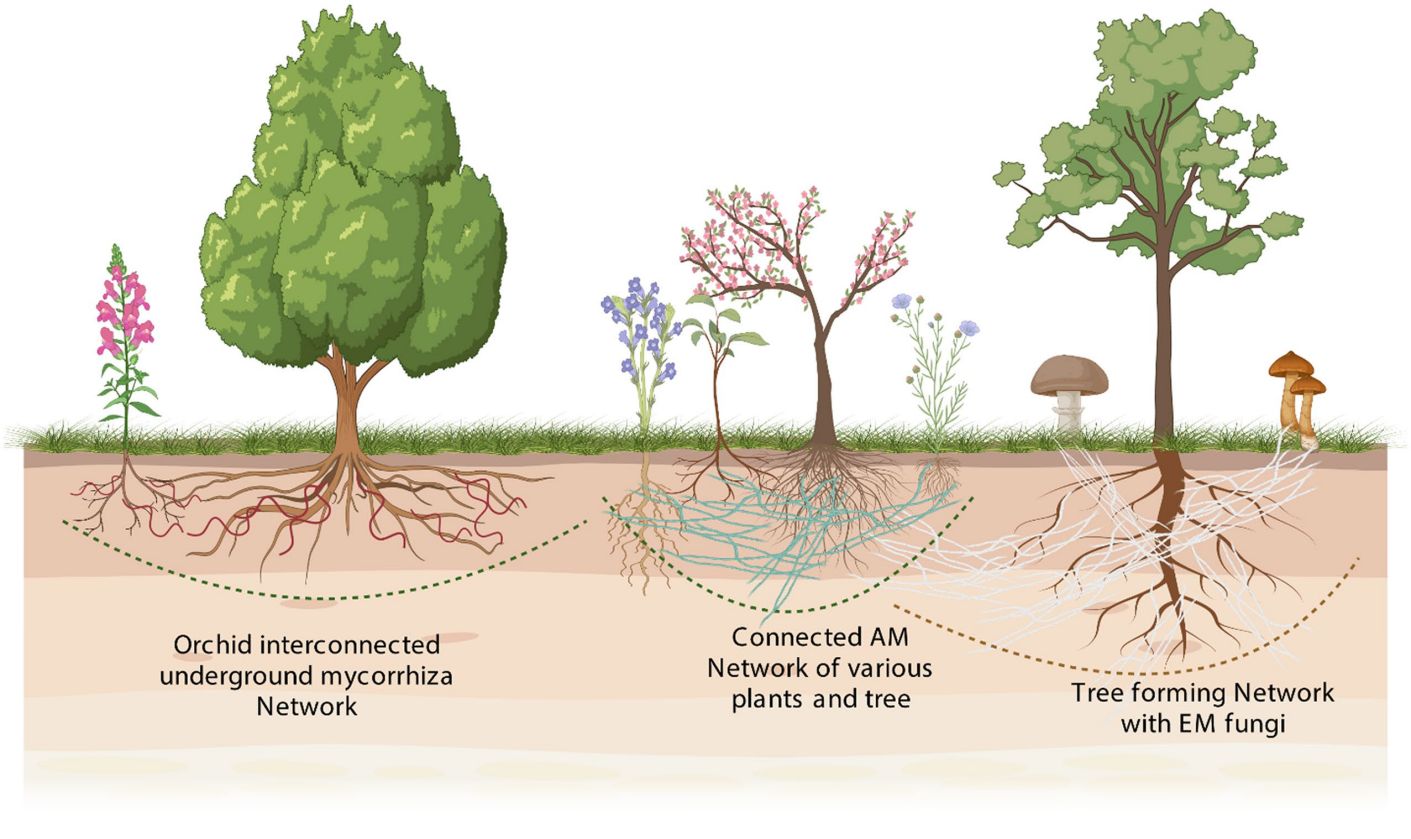
A hypothetical plant community graphic depicting plant species that have varying associations with different types of mycorrhizal fungi. In a hypothetical plant community, the graphic illustrates the diverse associations between different plant species and various types of mycorrhizal fungi. Mycorrhizal fungi are a highly diverse group of symbiotic fungi that form associations with plant roots, providing mutual benefits for both partners. While some plant species have evolved to form specific associations with particular types of mycorrhizal fungi, others may engage in associations with multiple fungal partners. These associations can vary in terms of their level of specificity and complexity. The graphic showcases the dynamic nature of these interactions, depicting how different plant species can have distinct associations with different types of mycorrhizal fungi. Such diversity in plant–fungi associations contributes to the resilience and functioning of plant communities by facilitating nutrient uptake, improving plant growth, and enhancing ecosystem sustainability.

Communication *via* multinucleated networks and dominant plant–microbe interactions are key components of the intricate relationships established between plants and microbes in natural environments. These co-interactions have evolved over millions of years, contributing to the growth, productivity, and overall fitness of both parties involved. These interactions can range from positive, such as enhancing stress tolerance, to negative, as observed in host–pathogen interactions, and are fundamental to the functioning of ecosystems. Classifying plant–microbe interactions based on their habitat specificity, such as their presence on leaf surfaces (phyllospheric), within plant tissues (endophytic), on root surfaces (rhizospheric), or as surface-dwelling organisms (epiphytic), helps to highlight the dense and intricate connections between plants and microbes that occur both above and below ground. In these complex relationships, microbes often engage in mutualistic interactions, where both parties derive mutual benefits, exemplifying the socialistic or capitalistic nature of these interactions (Huang et al., 2014; Panke-Buisse et al., 2015; Bandyopadhyay et al., 2017; Dutta and Bora, 2019; Liu et al., 2020). As previously mentioned, CMNs are underground networks created by the hyphae of mycorrhizal fungi, linking together multiple plants. CMNs allow plants to share resources and provide mutual benefits for all parties involved (Figure 3). The shared benefits afforded by these networks are significant in plant–microbe interactions (Song et al., 2010; Muneer et al., 2020). However, there are still many unknowns about the precise mechanisms through which these interactions occur. For example, it is not yet clear how the fungi are able to transport nutrients and other resources between plants or how they determine which plants to connect. Furthermore, there is still a need to research the impacts of CMNs on plant community dynamics, nutrient cycling, and ecosystem processes. Nevertheless, research has shown that CMNs are fundamental to plant–microbe interactions, enabling plants to cooperate and share resources in ways that are essential for their growth and survival (Chagas et al., 2018; Hassani et al., 2018; Rane et al., 2022).

The mutualistic interactions of plant communities show distinct growth responses to different AMF fungi through wide variation in host dependency and ability to regulate mycorrhizal colonization (Hoeksema et al., 2018; Davison et al., 2020). Among them, the rhizosphere is thought to be the most dynamic region where plant roots establish numerous rhizospheric interactions with countless microbial diversity for their growth and resistance against a variety of stresses (Bandyopadhyay et al., 2017).

Interactions of plants with rhizospheric microbes trigger the systemic plant defense response, which helps plants combat pathogens and pests (Vishwakarma et al., 2020). The plant immune responses, i.e., jasmonic acid, ethylene, and salicylic acid, are modulated by the interaction with rhizospheric microbes (Hu et al., 2018; Nascimento et al., 2018). CMNs play a super active role in plant–microbe interactions by inducing systemic resistance in plants against various disease-causing agents and several biotic and abiotic factors (Hoeksema et al., 2018; Davison et al., 2020). In the plant–microbe antagonism interaction, one interacting agent antagonizes the other by secreting cell wall-degrading enzymes such as cellulase, chitinase, proteinase, and lipase (Fellbaum et al., 2014). Through the formation of CMNs, these fungi release volatile organic chemicals, diffusible signal factor polyketides, and lipopeptides that elicit systemic resistance and defense in plants, which leads to the protection of plants from their pathogens (Song et al., 2014; Vahabi et al., 2018). Microbiome interactions with their host plants have a remarkable capacity to improve plant nutrition acquisition (Dellagi et al., 2020). They have the potential to transport, solubilize, and mineralize nutrients that are not freely available to the host. The availability of nutrients in the rhizosphere, such as nitrogen and phosphorous, to plants, is regulated by PGPRs, which thereby act as biofertilizers. As such, the utilization of beneficial microbes lessens the use of chemical-based synthetic pesticides and fertilizers in the agriculture industry (Alori et al., 2017). Mycorrhizal fungi, which are integral components of the CMNs, form symbiotic relationships with plant roots, providing them with improved access to soil nutrients. This is achieved through the ability of mycorrhizal fungi to extend the surface area of plant roots and by accessing nutrient sources that are not typically available to the plants through their own root systems (Etesami et al., 2021). In addition to the direct benefits provided by mycorrhizal fungi, the wider microbial community residing in the rhizosphere of plants also contributes to the acquisition of plant nutrients. Symbiotic interaction benefits both organisms, for example, the symbiotic interaction of leguminous plants and rhizobia (Davison et al., 2015; Lekberg and Waller, 2016), which helps not only in nitrogen fixation but also in nutrient acquisition. These microbial interactions may lead to the production of plant hormones and secondary metabolites that are involved in plant defense against pathogens and herbivores. Furthermore, CMNs have been shown to enhance the resistance of plants to insect herbivores by inducing systemic expression of defensive genes.

CMNs further contribute to the connectivity between plant communities and their rhizospheric microbial communities, facilitating the exchange of signals and information between plants and microbes (Unsicker et al., 2009). One of the key functions of CMNs is to facilitate communication between plant communities and their rhizospheric microbial communities, which includes the secretion of signaling molecules such as VOCs. These molecules can act as mediators between the plants and the microbial communities by influencing the behavior of the organisms (Ortíz-Castro et al., 2009; Abdul Hamid and Nadarajah, 2022). Recent studies have suggested that mycorrhizal fungi can release VOCs into the air, detecting them through their hyphae, and that these VOCs then act as a signal to neighboring plants to prime them for defense. These VOCs secreted by microorganisms are involved in the signaling processes between plants and microbes, playing a role in priming plants to express stress and defense responses (Canarini et al., 2019; Pang et al., 2021; Abdul Hamid and Nadarajah, 2022). Additionally, the presence of mycorrhizal fungi provides a favorable environment for the growth of rhizospheric microbes, leading to a more extensive network of interactions between the rhizosphere microbiome and the plant. These interactions can further mediate the production and exchange of signaling molecules, such as VOCs, between plants and microbes, influencing the behavior and survival of both (Ortíz-Castro et al., 2009; Delory et al., 2016). Microbial species secrete several signaling molecules, such as VOCs, which act as mediators between plants and microbial communities (Kanchiswamy et al., 2015).

## CMN functional characteristics for management interventions

Here, we list some of the important CMN functional characteristics that have the greatest potential to be impacted by management decisions. There is still considerable work to be done to test the importance of these findings in more realistic field-based conditions because the majority of studies on this subject have been carried out in simplified and controlled experimental settings.

## Nutrient distribution and retention

The puzzling question of “do plants incorporated into CMNs get more mineral nutrients than plants with solely an isolated community of mycorrhizal fungi?” is a critical query from a management perspective. Numerous studies have attempted to answer this question, but the results are conflicting, with some claiming that integration into CMNs has positive effects on nutrient uptake and biomass when compared to non-mycorrhizal controls or isolated mycorrhizal hosts (Yu et al., 2020), while others claim that it has negative effects (Janoušková et al., 2011; Merrild et al., 2013). *Andropogon gerardii* showed N inequality between large and small plants, highlighting the need to take into account the temporal dynamics of such responses on plant fitness, particularly in grazed ecosystems (Weremijewicz et al., 2016). For instance, in the short term, grazers may favor large stature plants at the expense of smaller stature plants but in the long run, smaller stature plants may benefit more nutrients from CMNs. Furthermore, even if CMNs have no effect on net nutrient uptake, they might have an impact on nutrient distribution, leading to more uniform performance across plant communities and potentially reducing fertilization-related pollution (Hamel et al., 1991). Based on resource disparities among the CMNs, AMF can control nutrient transfer (Welte, 2009; Whiteside et al., 2019), and the disparity appears to increase trading. For instance, P from various sources was translocated throughout the fungus in both directions, with a greater net movement from rich to poor areas (Whiteside et al., 2019). Walder et al. (2012) found that nutrient uptake and biomass gain were greater when two plant species (*Linum usitatissimum* L. and *Sorghum bicolor* L.) were interconnected by CMNs compared to when grown as monocultures and in the absence of mycorrhizal fungi. As a result, CMNs can alter trade with hosts not only by changing the amount of phosphorus and fungi trade (Whiteside et al., 2019) but also by altering the location of P transfer within the network (Jakobsen and Hammer, 2015; Noë and Kiers, 2018).

## Colonization

Establishment is crucial for plant seedling survival, and being promptly colonized by beneficial mycorrhizal fungi facilitates the colonization process. From a management standpoint, it is crucial to make sure that focal plants quickly become colonized by advantageous fungi. Incubation experiments consistently demonstrate that CMNs encourage seedling recruits to colonize quickly and uniformly (Marquez et al., 2018). However, from a field perspective, exploring such effects is more challenging because, first, it is rarely possible to determine with certainty whether a seedling is connected to larger CMNs, and second, it is challenging to quantify the mechanisms by which a seedling benefits (or does not benefit) from integration into CMNs. Despite these drawbacks, mesh cores continue to be a crucial tool for manipulating CMN presence in the field. Their application has demonstrated how CMNs encourage the establishment of new recruits in forests and grasslands (Liang et al., 2020). Through the use of a trenching technique, Booth and Hoeksema (2010) demonstrated how the connections to CMNs created by ECM had favorable effects on seedling establishment and benefited significantly from improved access to soil water. Furthermore, adult plants can help seedlings grow and absorb nutrients in challenging environments (like saline soils) by developing CMNs made by AM fungi. This highlights its potential use in the restoration of damaged ecosystems in saline soils (Yu et al., 2020). Following this, the use of CMNs could lessen the cost associated with AM fungi colonization in addition to their ability to efficiently colonize new hosts. Accordingly, there are no differences between potato plantlets that have recently been incorporated into CMNs and non-mycorrhizal plantlets in terms of, for example, P content or fresh weight, indicating that the cost of establishing AM fungi was likely lower for the incorporated plantlets and mostly borne by the donor plant (Gallou et al., 2012; Alaux et al., 2020). Because they have direct access to a significant reservoir of soil nutrients and water through the CMNs, seedlings may establish more readily within an existing mycorrhizal network.

## Resistance to pest and pathogen

Various below-ground signaling can occur in response to several biological stressors between plants infected with pests and non-infested neighbors through CMNs by acting as a conduit for interplant signaling (Vahabi et al., 2018; Song et al., 2019; Alaux et al., 2020). The fact that many of these studies were conducted on horticultural and agriculturally important plants like the broad bean, tomato, tobacco, and potato shows how CMNs may be used in agroecosystems. The idea of a sentinel plant, in which susceptible plants are checked periodically for indications of pest attack, could be developed further to take into account the ability of those plants to alert nearby neighbors (Figure 2). Contrastingly, the role of signaling in nature is still largely unknown, particularly with regard to the mechanism of signal transfer (Alaux et al., 2020) and the advantages to fungi’s overall fitness (Babikova et al., 2013). As already discussed, jasmonic acid (JA), ethylene (Song et al., 2014, 2019; Alaux et al., 2020), and salicylic acid (Song et al., 2010; Zhang et al., 2019) production have all been linked to plant responses. It’s interesting to note that the plant response or the priming of plant defense appears to only activate specific JA response components (Song et al., 2019), which may limit the potential cost of induced defense as a result of CMN signal transfer. Additional factors, such as the alleged signal relay between plants and potential connections between other CMNs, need to be studied in field settings in order to use this strategy effectively and reduce pest-related crop losses (Wipf et al., 2019).

## Conclusion and future directions

Plant–plant and plant–microbe interactions are extremely complex. More research is required to fully understand these interactions and to clarify how they can be used in agriculture for things like nutrient acquisition, improving disease resistance, and stress tolerance. To reveal the dynamic microbial colonization functions, advanced characterization techniques and large-scale experimental approaches are required. Numerous microbes have still not been fully characterized at the physiological and molecular levels. A fundamental question is whether CMNs have a greater (positive or negative) impact on plant performance and other ecosystem services than effects that result from mycorrhizal fungi alone. The absolute characterization of molecules facilitating beneficial microbes and inducing resistance against pathogens is a significant challenge because plant root exudates are made up of thousands of different substances. Numerous studies are still required to fully understand the variety, makeup, purposes, and mechanisms underlying the exchange of VOCs. We know very little about how AMF distributes infochemicals and nutrient resources within their CMNs or how plants compete with one another for the limited nutrient resources that are available for their CMNs. It is now possible to efficiently monitor microbial species that interact with plants due to the development of biotechnological tools. These may help us achieve our objective of maintaining the agricultural ecosystem’s sustainability. The benefits of CMNs within agroecosystems are, of course, not limited to the supply of plant nutrients. We identified a number of other important ecological functions of CMNs in soil. These include recycling of nutrients, prevention of nutrient losses, contribution to soil structure, food for other organisms, and mycorrhizal fungal networks acting as hyphal highways for bacterial dispersion. One of the most pressing concerns in agriculture is soil “health” and structure. CMNs maintain soil quality and health *via* three aspects: soil structure, plant physiology, and ecological interactions. AMF deposit glomalin between the outer hyphal walls and adjacent soil particles to form micro-aggregates and further macro-aggregates, thus forming the backbone for soil aggregation. CMNs hold huge significance for our planet and society and thus play an essential role in the formation and maintenance of global ecosystems. They also have great potential for exploitation to facilitate a variety of sustainability programs in agriculture, conservation, and restoration, particularly relevant in the context of global climate change and the depletion of natural resources. It is clear that CMNs are an essential component of ecosystem biodiversity and also deliver, through their roles in plant nutrition and protection, significant ecosystem services that have the potential to play an important role in sustainability agendas. However, significant knowledge gaps remain covering the multitude of interactions between plants, fungi, people, and the environment. This editorial provides an overview of the relevance and potential roles of mycorrhizal fungi toward achieving global goals in sustainability, conservation, and their significance within society, and highlights key directions for future research.

## Author contributions

All authors listed have made a substantial, direct, and intellectual contribution to the work and approved it for publication.
